# Metformin poisoning: A complex presentation

**DOI:** 10.4103/0019-5049.79890

**Published:** 2011

**Authors:** Manish Jagia, Salah Taqi, Mahmud Hanafi

**Affiliations:** Department of Anaesthesia and Intensive Care, Al Jahra Hospital, Kuwait

**Keywords:** Cardiac arrest, haemolytic anemia, lactic acidosis, metformin poisoning, pancreatitis

## Abstract

The objective of this case report is to highlight presentation, complications and treatment of metformin poisoning. Patient after ingestion of 45gms of metformin developed colicky abdominal pain, severe tachypnea and vomiting. He developed severe lactic acidosis, cardiac arrest, pancreatitis and hemolytic anemia which was treated with charcoal, sodium bicarbonate, early initiation of high volume continuous veno-venous hemofiltration and supportive therapy. Metformin poisoning is a rare presentation and we discuss course of events in the management of metformin poisoning and its associated complications.

## INTRODUCTION

Metformin is a biguanide oral hypoglycemic agent used for non-insulin dependent diabetes mellitus (NIDDM). Metformin poisoning can cause fatal complications like severe lactic acidosis, haemolytic anemia and pancreatitis. Early diagnosis can result in successful outcome. Here, we report a case having good recovery despite metformin induced complications and cardiac arrest.

## CASE REPORT

A 36-year-old man presented in the Emergency Department after ingestion of 45 g metformin. He presented with colicky abdominal pain, severe tachypnoea and vomiting. He had history of NIDDM and was on metformin since 6 months, with no other co-morbid conditions. On examination, his vital signs were as follows: heart rate 110/min, respiratory rate 40/min, warm extremities, blood pressure 140/80 mmHg and peripheral oxygen saturation on room air 97%. Systemic examination showed that he was conscious and oriented, tachypnoeic with bilateral equal air entry with no added sounds, guarding on abdominal examination due to abdominal pain. His initial blood sugar reading was 19 mmol/l and urine ketones were nil. Arterial blood gas (ABG) analysis showed severe metabolic acidosis (pH 6.85) with high lactate level (16 mmol/l) [Table 1]. Liver enzymes were mildly elevated and renal function test showed normal blood urea nitrogen but raised serum creatinine levels. Anion gap was 45 mmol/l. ECG and chest X-ray were normal. Ultrasound abdomen was normal with no signs of any collection intraperitoneally or splenomegaly. He was administered activated charcoal lavage and 8.4% sodium bicarbonate (50 ml) intravenously. The patient was transferred to ICU for high volume continuous veno-venous haemofiltration (CVVH). In ICU, ABG analysis showed persistent metabolic acidosis and 100 ml of 8.4% sodium bicarbonate was administered. After 45 minutes of admission to ICU, the patient complained of severe abdominal colic and vomited charcoal, followed by occurrence of sudden cardiac arrest. The patient was intubated and mechanically ventilated, and cardiopulmonary resuscitation (CPR) was initiated. The patient was successfully resuscitated with three cycles of CPR and sodium bicarbonate 50 ml (8.4%). The patient maintained his haemodynamics without any inotropic support. CVVH was started 4 hours after admission to hospital and continued over 24 hours. Serial ABG analysis showed persistent metabolic acidosis which improved to normal, 12 hours after the initiation of CVVH [Table 1]. Liver functions and renal functions deteriorated after cardiac arrest but improved over a period of days.

**Table 1:** Serial ABG analysis and lactate since admission

ABG/time	On admission	3 hours	6 hours	9 hours	16 hours	24 hours
pH	6.85	6.9	7.05	7.09	7.45	7.46
pO_2_(kPa)	16.7	15.4	14.2	12.4	9.2	10
pCO_2_ (kPa)	2.17	2.19	2.8	3.1	3.6	3.9
HCO_3_ (mmol/l)	5	5.2	6.4	7.0	18.8	20.6
BE	−27.9	−27.7	−24.5	−21.2	−4.2	−2.3
Lactate (normal: 0.5–2 mmol/l)	16	16	15	15	8.6	5.3

The patient in further course developed aspiration pneumonia, haemolytic anaemia and pancreatitis. His blood sugar was controlled by insulin. Aspiration pneumonia was treated with antibiotics, respiratory therapy and ventilation for 12 days before he was successfully extubated.

Haemoglobin dropped from 16.2 to 7.6 g/dl over 4 days with no sign of obvious bleeding. One unit of packed red blood cells was transfused. His stool for occult blood and Coomb’s test were negative. The serum iron, transferrin, vitamin B12 and folic acid were normal. Haemoglobin improved over 15 days with no more blood transfusion. Hepatitis screening for hepatitis B and C was unremarkable. Glucose-6-phosphate dehydrogenase (G6PD) level was normal. Peripheral smear revealed fragmented red blood cells (schistocytes).

On the day of admission, the patient had mildly raised lipase (72.4 U/l, normal 13–60 U/l) and amylase (188 U/l, normal 25–130 U/l). His lipase and amylase increased up to 591.1 U/l and 337 U/l, respectively, on 9^th^ day of admission. Other causes of pancreatitis like gallstone, alcoholism, hypertriglyceridemia, hypercalcaemia, autoimmune diseases, steroid or any drug intake were excluded. Computed tomography (CT) scan abdomen showed hypertrophic pancreas with no signs of necrosis. Supportive treatment in the form of antibiotics and fluids was given. The patient was shifted from ICU to ward after 16 days for further management.

## DISCUSSION

Metformin has good oral bioavailability with primarily renal excretion. Metformin decreases lactate metabolism by suppressing pyruvate carboxylase. It decreases glucose utilisation and increases lactate production by the hepatocytes. Therapeutic levels may be between 0.5 and 2 mg/l (0.5–1 mg/l in fasting state and 1–2 mg/l after meals). Metformin level assessment is important in toxicity; however, this facility is not available in our hospital.

Metformin induced lactic acidosis is treated by gastrointestinal (GI) decontamination, sodium bicarbonate, CVVH or haemodialysis. Metformin clearance by renal replacement therapies is debated due to high volume of distribution up to more than 3 l/kg (63–646 l/kg), as it is predominantly located intracellularly. Cardiac toxicity due to metabolic acidosis results in decreased cardiac contractility and can lead to cardiac arrest. This can be prevented by renal replacement therapies like CVVH or haemofiltration which helps in acute reduction of acidosis, electrolyte disturbances, and on prolongation causes metformin extraction from the intracellular compartment.[1]

Metformin impairs GI absorption of vitamin B12 and folic acid, but acute onset of haemolytic anaemia is unlikely. Haemolytic anaemia in our case was confirmed by anaemia, rise in lactate dehydrogenase (LDH), high reticulocyte counts and presence of schistocytes. Haptoglobin and fibrinogen levels were high due to cardiac arrest as they are acute phase reactants. The cause of haemolytic anaemia can be immune mediated[2] or due to G6PD deficiency caused by metformin.[3] The possible explanation for haemolysis in our case could be hepatitis A, which was excluded by history but not investigated in our case, or immune aetiology as the predictive value of a positive Coomb’s test for a patient with haemolytic anaemia is 83%.[4]

Metformin induced pancreatitis has rarely been reported as direct complication. It has been found in the presence of renal failure or multiple drug ingestion.[5]

In our case, pancreatitis was diagnosed serologically and by CT. It was managed by antibiotics and intravenous fluids for a couple of days, followed by resumption of enteral feed.

## CONCLUSION

Metformin poisoning associated with severe lactic acidosis, haemolytic anaemia and pancreatitis in a single case is very rare. We report this case to make clinicians aware of rare complications with metformin and consider early management with CVVH and supportive therapy to prevent fatal events like cardiac arrest.
